# Prognostic value of systemic immune-inflammation index for colorectal cancer: a systematic review and meta-analysis

**DOI:** 10.3389/fonc.2026.1616016

**Published:** 2026-02-05

**Authors:** Pengfei Wang, Huiying Jiang

**Affiliations:** Clinical Laboratory, Beijing Jingmei Group General Hospital, Beijing, China

**Keywords:** colorectal cancer, meta-analysis, overall survival, progression-free survival, systemic immune-inflammation index

## Abstract

**Background:**

Although the systemic immune-inflammation index (SII) has gained attention as a prognostic biomarker in colorectal cancer (CRC), existing studies report inconsistent findings due to methodological variability. This meta-analysis was conducted to clarify the prognostic value of SII in CRC.

**Methods:**

PubMed, Embase, the Cochrane Library and Web of Science were systematically searched for literature up to November 2025. The association between SII and clinical outcomes in CRC was identified. Studies that satisfying the inclusion and exclusion criteria were selected. Progression-free survival (PFS) and overall survival (OS) were the primary outcomes, which were presented by hazard ratios (HRs) with 95% confidence intervals (CIs). Heterogeneity and the stability of results were performed by subgroup and sensitivity analyses. Review Manager 5.4 and STATA 15.1 were conducted to analyze.

**Results:**

Thirty-five studies with 26812 cases were included. Elevated SII was associated with poorer OS (HR = 2.11, 95% CI: 1.73–2.57, p < 0.00001) and PFS significantly (HR = 2.16, 95% CI: 1.83–2.54, p < 0.00001). These associations remained consistent across subgroups stratified by geographic region, treatment modality, TNM stage, tumor location, and sample size. Sensitivity analyses confirmed the stability of the results. No significant publication bias exists for OS (p = 0.669) or PFS (p = 0.261) through Egger**’**s test.

**Conclusion:**

Elevated pre-treatment SII is associated with unfavorable survival and disease progression in CRC. However, the retrospective design of included studies and the substantial heterogeneity in SII cut-off values underscore the need for large-scale, prospective, multicenter investigations with standardized methodologies to validate these findings and establish optimal threshold values for clinical application.

**Systematic review registration:**

https://www.crd.york.ac.uk/prospero/, identifier CRD420251010606.

## 1 Introduction

Colorectal cancer (CRC) is a main global cause of cancer-related morbidity and mortality, exhibiting substantial prognostic variability even among patients classified within the same TNM stage. Although the TNM system forms the basis of survival assessment, its failure to account for inter-individual differences emphasizes the need for supplementary biomarkers to refine risk stratification. Systemic inflammation, a hallmark of tumor progression, plays a critical role in CRC pathogenesis. Inflammatory biomarkers such as the neutrophil-to-lymphocyte ratio (NLR) and platelet-to-lymphocyte ratio (PLR) have shown prognostic value (13). The systemic immune-inflammation index (SII), calculated as platelet × neutrophil/lymphocyte, has emerged as a reliable indicator of immune-inflammatory status (1). Initially validated in hepatocellular carcinoma, its prognostic relevance in CRC has yet to be fully established.

Recent evidence underscores the prognostic significance of the SII in CRC. In metastatic CRC, a low SII is associated with prolonged progression-free and overall survival among patients receiving first-line chemotherapy (2, 3), whereas an elevated preoperative SII independently predicts inferior overall and disease-free survival following curative resection (1). Subgroup analyses indicate that the SII more accurately differentiates TNM stage-specific survival than the NLR or PLR and maintains prognostic relevance in stage II colorectal cancer when stratified by tumor sidedness (1, 4). Dynamic alterations in the systemic immune-inflammation index change (ΔSII) following resection offer superior prognostic precision compared to static measurements, with elevated ΔSII associated with a 4.3-fold increase in mortality risk (5). SII also retains prognostic relevance in distinct subgroups, including patients with liver metastases (7), obstructive colorectal cancer (14), and microsatellite instability-high tumors treated with immunotherapy (15).

Inconsistencies persist due to methodological heterogeneity. The prognostic significance of the SII varies by K-ras genotype, demonstrating relevance only in wild-type metastatic CRC (6), and its optimal cut-off values differ between colon (535) and rectal liver metastases (792) (7). Although meta-analyses report pooled HR for overall (HR = 1.86) and disease-free survival (HR = 2.03) (8) in digestive malignancies, CRC-specific evaluations remain limited. A recent meta-analysis by Tan et al. included 27 studies with a literature search updated to March 2024 (9). However, variations in study design, endpoint definitions, and patient characteristics—such as treatment strategies, geographic distribution, and inflammatory thresholds—continue to constrain clinical applicability.

This meta-analysis investigates the prognostic utility of the SII in CRC, addressing heterogeneity through comprehensive subgroup analyses stratified by disease stage, treatment modality, tumor location, TNM stage, and biomarker dynamics. Drawing on data from 35 studies comprising 26812 patients, it evaluates the role of SII in enhancing survival prediction and identifying high-risk subgroups to guide individualized treatment strategies.

## 2 Materials and methods

### 2.1 Literature search

This study was conducted in accordance with PRISMA 2020 guidelines and was prospectively registered in PROSPERO (CRD420251010606) (10). Two investigators (WPF and JHY) independently performed literature searching. Searching terms as following: **“**Colorectal, Neoplasm,**” “**Neoplasm, Colorectal,**” “**Colorectal Tumors,**” “**Colorectal Tumor,**” “**Tumor, Colorectal,**” “**Tumors, Colorectal,**” “**Neoplasms, Colorectal,**” “**Colorectal Cancer,**” “**Cancer, Colorectal,**” “**Cancers, Colorectal,**” “**Colorectal Cancers,**” “**Colorectal Carcinoma,**” “**Carcinoma, Colorectal,**” “**Carcinomas, Colorectal,**” “**Colorectal Carcinomas,**” “**Systemic Immune-Inflammation Index,**” “**systemic immune inflammation index.**”** The detailed literature searching strategy was depicted in **Supplementary Table S1**.

### 2.2 Study selection

Inclusion criteria: 1) CRC confirmed by pathological diagnosis; 2) treatment involving surgery, radiochemotherapy, neoadjuvant radiochemotherapy, or a combination thereof; 3) evaluation of the prognostic significance of the SII for PFS or OS; 4) availability of HRs with 95% confidence intervals (CIs), either reported or derivable from data; 5) stratification into high and low SII groups based on predefined cut-off values; 6) publication as a full-text article.

Exclusion criteria were as follows: 1) reviews, commentaries, conference abstracts, case reports, or letters; 2) insufficient data to estimate HRs and CIs; 3) lack of survival outcomes; and 4) duplicate or overlapping datasets.

WPF and JHY screened titles and abstracts, reviewed full texts, and assessed study eligibility independently. Discrepancies were resolved through discussion.

### 2.3 Data extraction

WPF and JHY extracted the data independently, with discrepancies resolved through consensus. The first author, publication year, study location, study design, sample size, patient age, study duration, treatment modality, type of immune checkpoint inhibitor, timing of detection, cut-off value, follow-up period, and HRs with 95% CIs for OS and PFS were extracted. When studies simultaneously reported both univariate and multivariate analysis results, multivariate analysis results were preferentially extracted. For studies reporting **“**Low SII/High SII**”** comparisons, HRs and CIs were transformed by calculating reciprocals and inverting confidence limits to ensure consistent **“**High SII/Low SII**”** comparisons across analyses.

### 2.4 Quality assessment

Study quality was evaluated by Newcastle-Ottawa Scale (NOS), which assesses selection, comparability, and outcome domains, with a maximum score of nine. High quality studies score 7-9. Detailed scoring criteria and reasons for each assessment are provided in **Supplementary Table S2**.

### 2.5 Statistical analysis

Pooled HRs with 95% CIs were computed to estimate the prognostic significance of the SII in CRC. Cochran**’**s Q test and Higgins**’** I² statistic were used to evaluate the heterogeneity (11). Heterogeneity was deemed significant when the Q-test P-value was < 0.1 or I² exceeded 50%. Data synthesis was conducted using a random-effects model. Sensitivity and subgroup analyses were performed to assess result robustness and identify heterogeneity sources. Funnel plots, Egger**’**s tests were used to examine the publication bias. A two-sided P-value < 0.05 was considered statistically significant. All analyses were conducted using STATA 15.0 and Review Manager 5.4.

## 3 Results

### 3.1 Study characteristics

**Figure 1:**
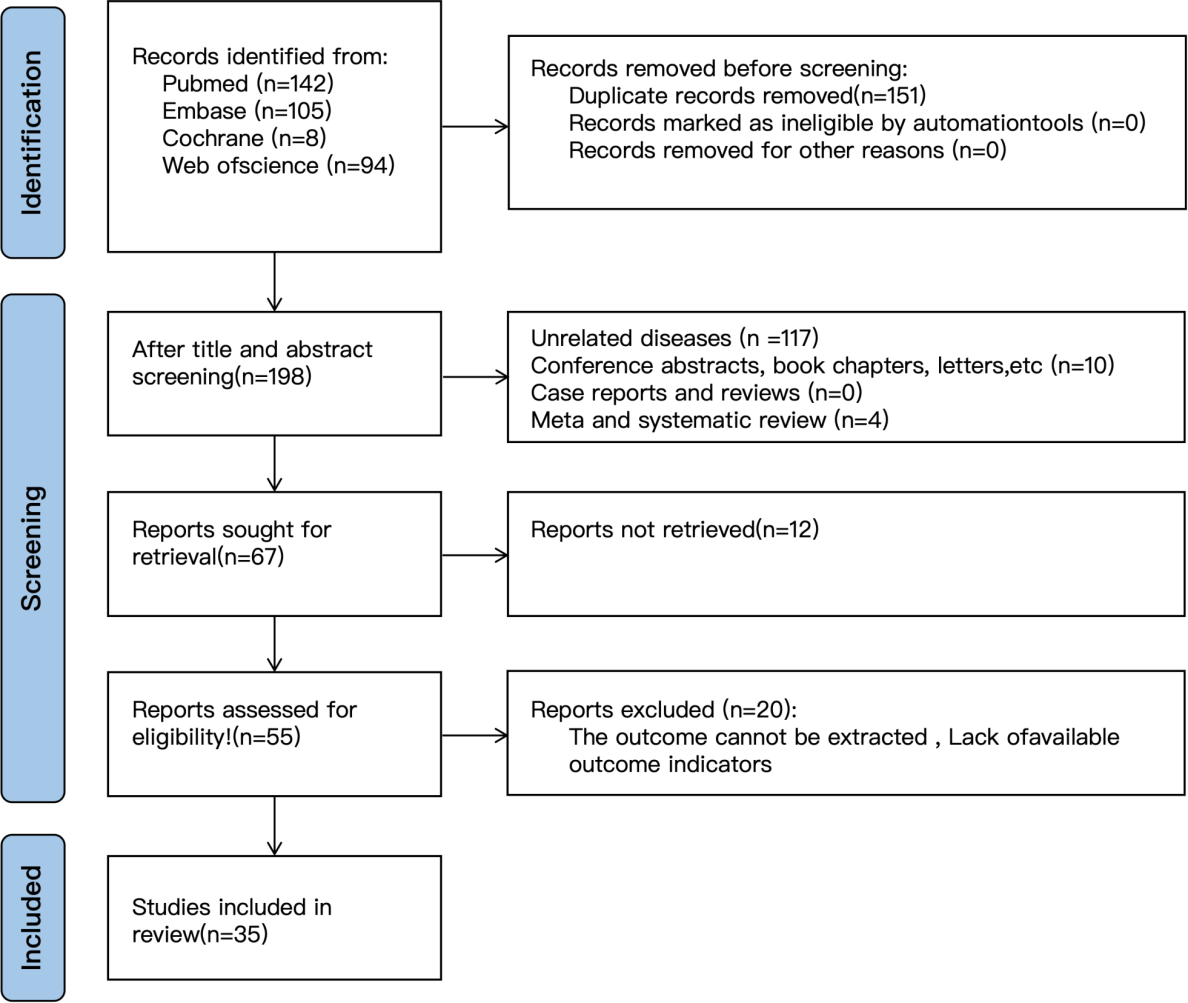
Flow chart of literature screening.

A total of 349 articles were retrieved: PubMed (n=142), Embase (n=105), Cochrane Library (n=8), and Web of Science (n=94). After removing 151 duplicates, 198 records remained. Screening of titles and abstracts excluded 131 records, comprising 117 unrelated diseases, 10 conference abstracts, book chapters, or letters, and 4 meta-analyses or systematic reviews. Sixty-seven articles were identified as potentially eligible and retrieved for full-text review. Twelve were unavailable, leaving 55 for further assessment. Twenty were excluded due to insufficient data or absence of outcome indicators. Ultimately, 35 studies comprising 26812 patients were included (**Figure 1**) (1–6, 8–40).

Studies were conducted across multiple countries, with the majority from Asia (China, Japan), followed by Europe (Italy, Hungary, Spain) and North America (USA). Four publications each included two cohorts, resulting in a total of 40 cohorts for analysis. Among these, 38 were retrospective and 2 were prospective. All cohort studies were published in English between 2016 and 2025. Study populations included patients with metastatic CRC, those undergoing surgical resection, and individuals receiving surgery, chemotherapy, targeted therapy, or immunotherapy. The SII was primarily measured at baseline or preoperatively, with some studies evaluating postoperative levels. Cut-off values varied substantially across studies, ranging from 340 to 1505. Detailed characteristics of studies are presented in **Table 1**.

**Table 1:** Basic characteristics of the included literature.

Author	Study period	Region	Study design	Population	Therapeutic approach	Test time	Sample size	Male	Female	Mean/Median age	Mean/Median BMI	Mean/Median tumor size	TNM stage	SII cut-off	Outcome	Quality score
CasadeiGardini 2020	2007-2012	Italy	Retrospective	Metastatic colorectal cancer patients	First-line chemotherapy ± bevacizumab (CT ± B)	Baseline	131	78	53	67	NA	NA	IV	6068	OS,PFS	8
Chang 2023	2004-2014	China	Retrospective	Patients who underwent colorectal cancer resection	Colorectal cancer resection ± adjuvant therapy	Within 14 days before surgery	768	416	352	70	NA	NA	II	616	OS	8
Chen 2017	1994-2010	China	Retrospective	Colorectal cancer (CRC) patients	Colorectal cancer resection ± adjuvant chemotherapy	Before surgery	1383	808	575	60	NA	5	I-IV	340	OS,PFS	7
Chen 2020	2010-2015	China	Retrospective	Colorectal cancer (CRC) patients	Surgical resection ± adjuvant treatment	7 days before surgery	206	108	98	56.8	NA	NA	I-IV	127.7	OS,PFS	7
Deng 2021	2006-2016	China	Retrospective	Colorectal liver metastasis (CRLM) patients	Radical resection ± perioperative chemotherapy	7 days before surgery	283	187	96	57	NA	2.4	I-IV	0.0135	OS,PFS	8
Gao 2022	2014-2020	China	Retrospective	Patients with digestive system tumors (including esophageal, gastric, and colorectal cancer)	Surgical resection	Before surgery	8384	5844	2540	NA	NA	NA	I-IV	550	OS,PFS	7
Huang 2020	2013-2017	China	Retrospective	Patients with colorectal cancer who underwent radical surgery	Radical surgery	Before surgery	1279	763	516	28-75	NA	NA	I-III	340	OS,PFS	9
Jiang 2019	2010-2017	China	Retrospective	Patients with metastatic colorectal cancer (mCRC) and RAS wild-type (WT) who received first-line cetuximab combined with chemotherapy	First-line chemotherapy + cetuximab	Within 3 days before treatment	102	72	30	NA	NA	NA	mCRC	660.55	OS,PFS	8
Jin 2022	2012-2015	China	Retrospective	Patients with stage I colorectal cancer (CRC) who underwent radical surgery	Radical surgery	Within 1 week before surgery	476	259	217	60.8	NA	NA	I(pT1-2N0)	540.262	OS	8
Li 2020	2016-2019	China	Retrospective	Colorectal cancer (CRC) patients	Surgery, neoadjuvant chemoradiotherapy, chemotherapy, or no surgery	Before treatment	3919	NA	NA	NA	NA	NA	NA	340-1505	OS,PFS	8
Li 2025	2010-2020	Jingdezhen, Jiangxi Province, China	Retrospective	Colorectal cancer (CRC) patients	Radical resection	Within 7 days preoperatively	324	183 cases (56.48%)	141 cases (43.52%)	62.60 ± 12.49	NA	5	I-III	532.985	OS	8
Miyamoto 2023	2005-2019	Japan	Retrospective	Patients with metastatic colorectal cancer who received first-line systemic chemotherapy	First-line systemic chemotherapy	2 weeks before chemotherapy	272	141	131	63	22.6	NA	II-III	640	OS	8
Moro-Valdezate 2025a	2011-2019	Valencia, Spain	Observational	Stage I–III colorectal cancer patients undergoing curative resection (age ≥18)	Curative surgical resection (laparoscopic or open approach)	Preoperative peripheral blood sampled within 1 month before surgery	764	465 (60.9%)	299 (39.1%)	65	27.68 kg/m²	NA	I-III	919.48	PFS	8
Moro-Valdezate 2025b	2011-2019	Valencia, Spain	Observational	Stage I–III colorectal cancer patients undergoing curative resection (age ≥18)	Curative surgical resection (laparoscopic or open approach)	Preoperative peripheral blood sampled within 1 month before surgery	764	465 (60.9%)	299 (39.1%)	65	27.68 kg/m²	NA	I-III	1401.01	PFS	8
Nakamoto 2023	2012-2017	Japan	Retrospective	CRC patients who underwent radical resection	Radical resection	Preoperative	118	72	46	70(34-94)	22.2	NA	0-III	598	PFS	8
Passardi 2016	2007-2012	Italy	Prospective	Patients with metastatic colorectal cancer	First-line chemotherapy ± bevacizumab (FOLFIRI/FOLFOX4 ± B)	Baseline (before systemic treatment)	289	103	65	65	NA	NA	IV	730	OS,PFS	8
Passardi 2023	2016-2019	Italy	Prospective	Patients with metastatic colorectal cancer (mCRC) who received first-line chemotherapy combined with bevacizumab	First-line chemotherapy + bevacizumab (FOLFIRI/FOLFOX4/CAPIRI/CAPOX + B)	At baseline and before each treatment cycle	182	103	65	65	NA	NA	IV	730	OS,PFS	9
Polk 2022	2001-2018	Hungary	Retrospective	Colon cancer liver metastasis (CLM) patients	Surgery, CT, targeted therapy	24–48 hours before surgery	67	36	31	65(38-80)	NA	NA	NA	535	PFS	8
Sato 2023	2013-2020	Japan	Retrospective	Obstructive CRC (OCRC) patients treated with self-expanding metal stents (SEMS) as palliative or bridge-to-surgery	Palliative treatment, bridge-to-surgery	Preoperative	92	50	36	71	21.6	NA	I-III	597	PFS	8
Şentürk 2025a	2015-2023	Turkey (Sakarya Training and Research Hospital)	Retrospective	Rectal cancer patients	Low anterior resection; Neoadjuvant therapy (34.22%); Adjuvant therapy (82.57%) with FOLFOX/FOLFIRI regimens	Preoperative (baseline)	637	407 (63.9%)	230 (36.1%)	63.55 ± 12.49	NA	5	T-stage: I -IV N-stage: N0-N2	846.259	OS	8
Şentürk 2025b	2015-2023	Turkey (Sakarya Training and Research Hospital)	Retrospective	Rectal cancer patients	Low anterior resection; Neoadjuvant therapy (34.22%); Adjuvant therapy (82.57%) with FOLFOX/FOLFIRI regimens	Preoperative (baseline)	637	407 (63.9%)	230 (36.1%)	63.55 ± 12.49	NA	5	T-stage: I -IV N-stage: N0-N2	846.259	OS	8
Şentürk 2025c	2015-2023	Turkey (Sakarya Training and Research Hospital)	Retrospective	Rectal cancer patients	Low anterior resection; Neoadjuvant therapy (34.22%); Adjuvant therapy (82.57%) with FOLFOX/FOLFIRI regimens	Preoperative (baseline)	637	407 (63.9%)	230 (36.1%)	63.55 ± 12.49	NA	5	T-stage: I -IV N-stage: N0-N2	846.259	OS	8
Su 2025	2015-2023	China (Suzhou/Changzhou, Jiangsu Province)	Retrospective	Patients with advanced left-sided CRC (initially unresectable or postoperative recurrent stage IV) receiving first-line CAPEOX ± bevacizumab	First-line chemotherapy: CAPEOX (capecitabine + oxaliplatin) ± bevacizumab	Blood samples obtained within 1 month prior to starting first-line chemotherapy	231	162 (70.1%)	69 (29.9%)	65	NA	NA	IV	1,424.80	OS	8
Sun 2024a	2018-2020	China	Retrospective	Rectal cancer patients undergoing radical surgery	Laparoscopic radical surgery	Within 7 days before surgery	292	173	119	71	21.6	NA	I-III	449.325	PFS	8
Sun 2024b	2018-2020	China	Retrospective	Rectal cancer patients undergoing radical surgery	Laparoscopic radical surgery	Within 21–56 days after surgery	292	173	119	71	21.6	NA	NA	568.13	PFS	8
Xiang 2023	2013-2017	China	Retrospective	Patients under 50 years old diagnosed with colorectal cancer who underwent radical resection	Radical resection	Before surgery or within 1 week before surgery	236	143	93	45	22.9	NA	I-III	637.6	OS	9
Xie 2018	2009-2014	China	Retrospective	Patients with stage IV metastatic colorectal cancer (mCRC)	Radical surgery	Before surgery	240	157	83	59(18-90)	NA	NA	IV	649.45	OS	8
Xie 2020	2012-2014	China	Retrospective	Colorectal cancer patients	Surgery ± adjuvant chemotherapy	Before surgery	662	408	254	59	NA	5.0 ± 2.2	TNM	649.4	OS,PFS	8
Yan 2020	1997-2013	China	Retrospective	Patients with synchronous colorectal peritoneal cancer (SCRPC) who underwent cytoreductive surgery (CRS) and systemic chemotherapy (CT)	Cytoreductive surgery + systemic chemotherapy	Before surgery or within 1 week before surgery	103	67	36	NA	NA	NA	TNM	410	OS	8
Yang 2017	2009-2016	China	Retrospective	Patients with wild-type RAS metastatic colorectal cancer (mCRC) who received cetuximab treatment	First-line chemotherapy + cetuximab	Within 10 days before cetuximab administration	95	58	37	56	NA	NA	M1a\M1b	460.66	OS,PFS	8
Yang 2018	2010-2017	China	Retrospective	Patients with colorectal cancer who received neoadjuvant chemoradiotherapy (CRT)	Neoadjuvant chemoradiotherapy (CRT)	Within 4 weeks before CRT	98	59	39	53	NA	NA	TNM	437.72	OS,PFS	8
Yang 2019	2009-2015	China	Retrospective	Stage III/IV CRC patients receiving adjuvant chemoradiotherapy	Adjuvant chemoradiotherapy (CRT)	Within 2 weeks before radiotherapy	220	87	133	56 (23–78)	NA	NA	III-IV	534.94	PFS	8
Yatabe 2020a	2010-2014	Japan	Retrospective	Patients who underwent colorectal cancer resection	Surgical resection	Within 3 weeks before surgery	733	463	270	66(58-74)	NA	NA	0-IV	NA	OS	8
Yatabe 2020b	2010-2014	Japan	Retrospective	Patients with colorectal cancer (CRC)	Colorectal cancer resection ± preoperative radiotherapy/chemotherapy	Within 3 weeks before surgery	733	463	270	66	NA	NA	0-IV	NA	OS	8
Yi 2023	2017-2022	China	Retrospective	Patients with MSI-H metastatic colorectal cancer (mCRC) who received anti-PD-1 treatment	Anti-PD-1 treatment ± chemotherapy/anti-angiogenesis therapy	Within 1 week before surgery	75	48	27	47(23-84)	NA	NA	IV	409.6	OS,PFS	8
Young 2023	2014-2019	USA	Retrospective	Patients with metastatic colorectal cancer (mCRC) who received transarterial radioembolization (TARE)	Transarterial radioembolization (TARE)	30 days after surgery	41	21	20	61.4 ± 8.2	NA	NA	NA	409.6	OS	8
Yuan 2025	2020-2022	Chongqing, China (First Affiliated Hospital of Army Medical University)	Retrospective	pMMR advanced colorectal cancer patients (confirmed by immunohistochemistry, clinical stage IV)	Camrelizumab (anti-PD-1) combined with bevacizumab (anti-VEGF) targeted therapy	Baseline (before immunotherapy initiation)	216	120 (55.6%)	96 (44.4%)	57(25-83)	25 kg/m²	NA	IV	663.9	OS,PFS	8
Zeynelgil 2025	2020-2024	Türkiye	Retrospective	Patients with metastatic colorectal cancer	Chemotherapy with or without biological agents; metastasectomy for selected patients	Pre-treatment, before the first chemotherapy	155	99	56	60 (24-83)	NA	NA	IV	835.96	OS	8
Zhang and Miao 2023	2019-2023	China	Retrospective	Patients who underwent colorectal cancer resection	Radical resection for colorectal cancer	Before surgery	160	62	41	61.4 ± 8.2	NA	NA	TNM	513.53	OS	8
Zhou 2018	2007-2015	China	Retrospective	Patients who underwent colorectal cancer resection	Colorectal cancer resection ± preoperative/postoperative adjuvant treatment	Before surgery	516	331	185	58.4	NA	NA	I-IV	568.69	OS,PFS	8

### 3.2 Study quality

Study quality was evaluated using the NOS, with scores of 7–9 reflecting high methodological rigor (**Supplementary Table S2**). The mean NOS score was 7.8, indicating overall good quality of included studies.

### 3.3 Meta-analysis results

#### 3.3.1 SII and OS

**Figure 2:**
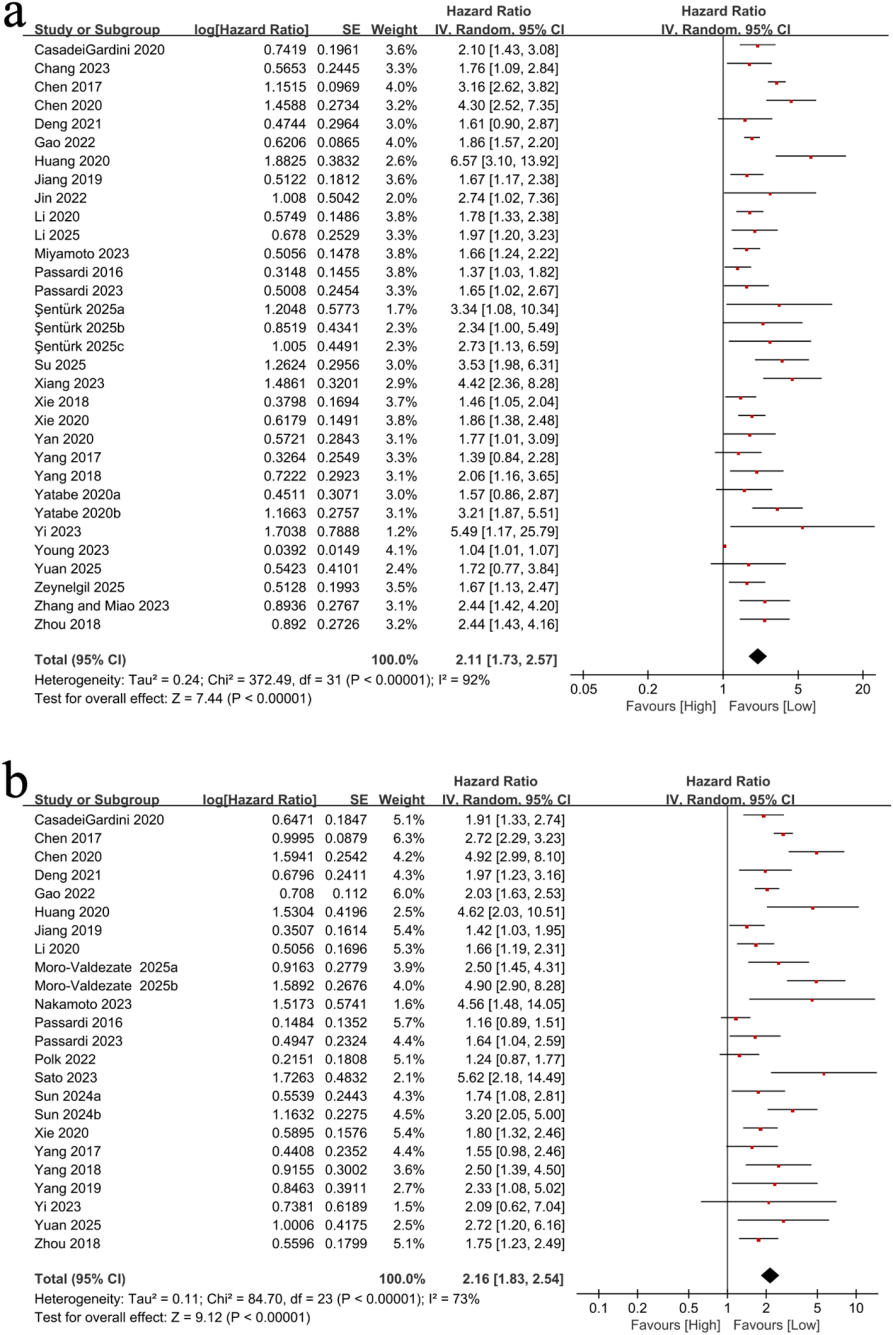
Forest plots of the association between the systemic immune-inflammation index (SII) and survival outcomes: **(a)** overall survival (OS); **(b)** progression-free survival (PFS).

We evaluated the association between SII and OS using 32 cohort studies (25,366 participants). Given the substantial heterogeneity (I² = 92%, p < 0.00001; **Figure 2A**), a random-effects model was applied. Elevated SII was significantly associated with worse OS (HR = 2.11, 95% CI: 1.73–2.57; p < 0.00001; **Figure 2A**). Prespecified subgroup analyses showed broadly consistent associations across study design, population characteristics, tumor location, treatment modality, timing of SII measurement, geographic region, and sample size (**Table 2**). Notably, the association appeared strongest in stage III disease (HR = 3.65, 95% CI: 1.98–6.91), and post-treatment SII showed a numerically stronger association than baseline SII (post-treatment: HR = 2.44 vs baseline: HR = 1.98), suggesting potential prognostic value of dynamic inflammatory status during treatment.

**Table 2:** Subgroup analysis results for overall survival (OS).

Subgroup	Number of studies	Pooled HR (95% CI)	I² (%)	P-value
Overall	32	2.11 (1.73–2.57)	92	<0.00001
Study design				
Retrospective	30	2.05 (1.67–2.52)	92	<0.00001
Prospective	2	1.89 (1.45–2.47)	45	<0.00001
TNM stage				
Stage I–II	8	2.31 (1.65–3.24)	78	<0.00001
Stage III	4	3.65 (1.98–6.91)	85	<0.00001
Stage IV/Metastatic	19	2.47 (1.34–4.84)	93	<0.00001
Tumor location				
Colon	12	2.18 (1.67–2.85)	89	<0.00001
Rectal	8	1.96 (1.45–2.65)	85	<0.00001
Mixed/Colorectal	12	2.05 (1.62–2.59)	91	<0.00001
Treatment modality				
Chemotherapy alone	14	2.01 (1.52–2.66)	90	<0.00001
Chemo + Bevacizumab	6	1.78 (1.23–2.58)	85	0.002
Surgical resection	16	2.24 (1.78–2.82)	93	<0.00001
Timing of measurement				
Baseline	24	1.98 (1.62–2.43)	91	<0.00001
Post-treatment	8	2.44 (1.43–4.17)	89	0.001
Geographic region				
Asia	26	2.12 (1.70–2.64)	92	<0.00001
Europe/America	6	1.87 (1.45–2.42)	88	<0.00001
Age group				
≥ro years	12	2.34 (1.78–3.08)	88	<0.00001
<65 years	20	1.89 (1.52–2.35)	92	<0.00001

Meta-regression suggested a stronger prognostic effect in older populations (P = 0.041). Additionally, *post-hoc* meta-regression analyses indicated that the reported SII cut-off value was not a significant moderator of the pooled OS effect estimate (p = 0.938). The findings were consistent when using log-transformed cut-off values (p = 0.889) (**Table 3**).

**Table 3:** *Post-hoc* meta-regression of SII cut-off value as a moderator for OS and PFS.

Outcome	Moderator	β	95% CI	P value	Studies(k)
OS	Cut-off (continuous)	–0.00001	–0.00014–0.00013	0.94	28
OS	ln(Cut-off)	0.006	–0.072–0.083	0.89	28
PFS	Cut-off (continuous)	–0.00002	–0.00017–0.00012	0.75	23
PFS	ln(Cut-off)	–0.002	–0.080–0.077	0.97	23

#### 3.3.2 SII and PFS

We investigated the association between SII and PFS across 24 cohort studies (19,402 participants). Substantial heterogeneity was observed (I² = 73%, p < 0.00001), and a random-effects model was used. Elevated SII was significantly associated with shorter PFS (HR = 2.16, 95% CI: 1.83–2.54; p < 0.00001; **Figure 2B**). Subgroup analyses demonstrated generally consistent findings across key strata, including metastatic status, TNM stage, tumor location, timing of measurement, and geographic region (**Table 4**). The association was robust in retrospective studies, whereas it did not reach statistical significance in the limited number of prospective cohorts, potentially reflecting smaller sample size and reduced power. *Post-hoc* meta-regression analyses similarly showed that the reported SII cut-off value did not significantly moderate the pooled PFS effect estimate (p = 0.746). Consistent results were obtained using log-transformed cut-off values (p = 0.968) (**Table 3**).

**Table 4:** Subgroup analysis results for progression-free survival (PFS).

Subgroup	Number of studies	Pooled HR (95% CI)	I² (%)	P-value
Overall	24	2.16 (1.83–2.54)	73	<0.00001
Study design				
Retrospective	22	2.08 (1.74–2.49)	74	<0.00001
Prospective	2	1.28 (0.98–1.67)	45	0.07
TNM stage				
Stage III	6	3.64 (1.65–8.60)	89	0.002
Stage IV/Metastatic	12	2.23 (1.38–4.01)	78	0.001
Mixed stages	6	1.97 (1.31–2.98)	65	0.001
Tumor location				
Colon	10	2.24 (1.78–2.82)	78	<0.00001
Rectal	6	1.89 (1.42–2.52)	69	<0.00001
Mixed/Colorectal	8	2.08 (1.62–2.67)	72	<0.00001
Treatment modality				
Chemotherapy alone	10	2.01 (1.52–2.66)	75	<0.00001
Chemo + Bevacizumab	4	1.41 (0.97–2.06)	68	0.07
Surgical resection	12	2.18 (1.74–2.73)	74	<0.00001
Timing of measurement				
Baseline	18	2.01 (1.65–2.45)	74	<0.00001
Post-treatment	6	2.18 (1.45–3.28)	72	<0.00001
Geographic region				
Asia	20	2.19 (1.82–2.63)	74	<0.00001
Europe/America	4	1.89 (1.42–2.52)	68	<0.00001

### 3.4 Sensitivity analysis

**Figure 3:**
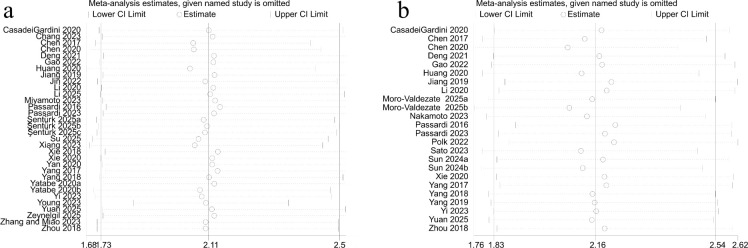
Sensitivity analyses of the association between the systemic immune-inflammation index (SII) and survival outcomes: **(a)** overall survival (OS); **(b)** progression-free survival (PFS).

Sensitivity analysis confirmed the stability of the baseline SII findings, as effect sizes remained consistent when individual studies were sequentially excluded. No single study exerted a disproportionate influence on the results for OS (**Figure 3A**) or PFS (**Figure 3B**), supporting the overall reliability of the analysis.

### 3.5 Publication bias

**Figure 4:**
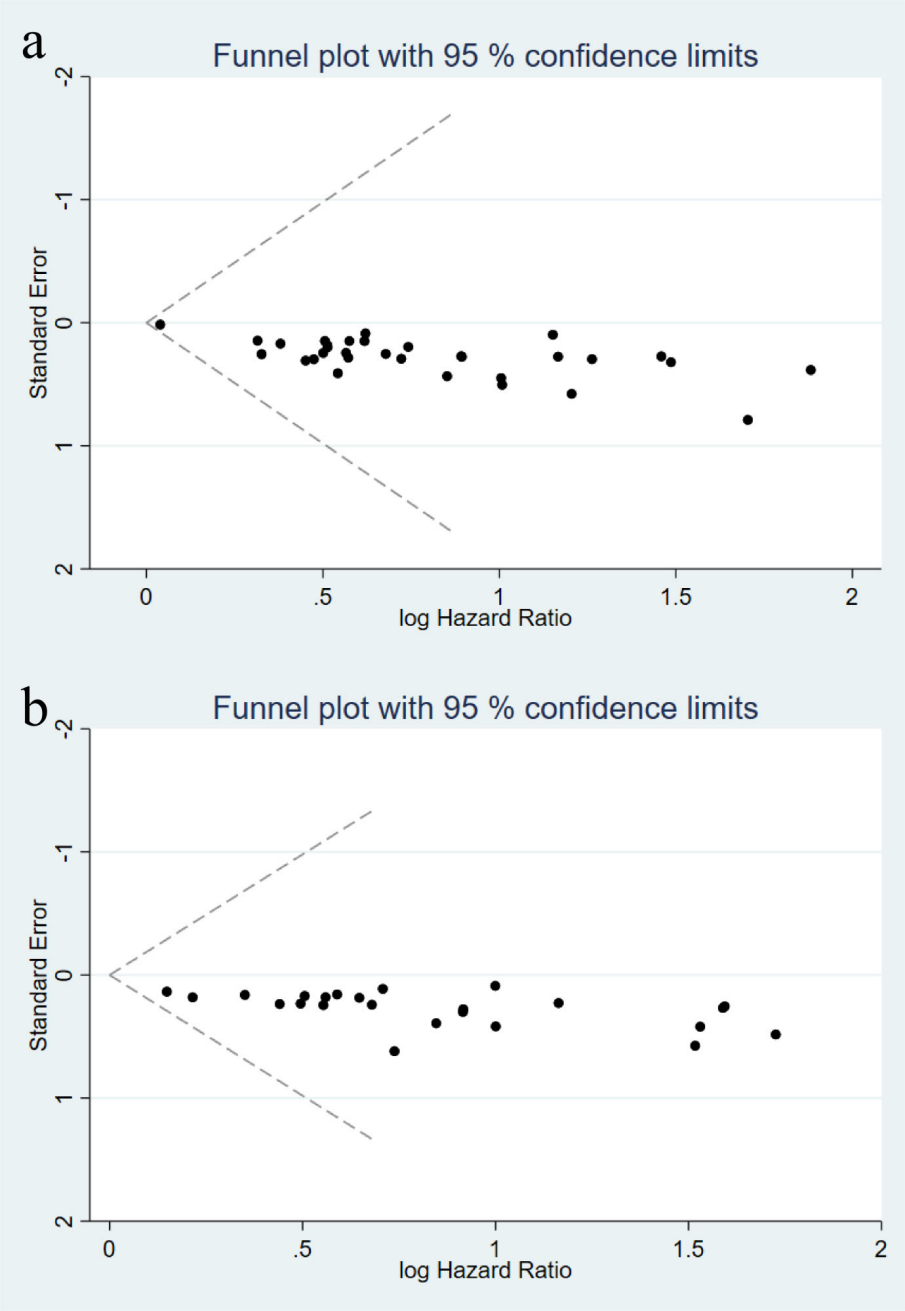
Funnel plots for assessing publication bias in the meta-analysis of the systemic immune-inflammation index (SII) and survival outcomes: **(a)** overall survival (OS); **(b)** progression-free survival (PFS).

Publication bias was assessed using funnel plots with pseudo 95% confidence limits and Egger’s regression test. Visual inspection of the funnel plots for OS and PFS suggested an approximately symmetric distribution of studies within the 95% confidence boundaries (**Figures 4A, B**), with no obvious small-study effects. Consistently, Egger’s test was not statistically significant for either OS (p = 0.669) or PFS (p = 0.261), indicating no evidence of publication bias.

## 4 Discussion

Systemic inflammation contributes to tumor progression by promoting genetic mutations, genomic instability, epigenetic alterations, tumor metastasis, and cancer cell proliferation (12). Blood-derived markers, recognized for their accessibility and reproducibility, serve as robust indicators of systemic inflammation and patient prognosis (8, 13). The SII, defined as platelet count × neutrophil count/lymphocyte count, represents the interplay between host inflammatory status and tumor progression (1). However, studies evaluating its prognostic utility in CRC have yielded inconsistent results, likely due to variability in metastatic status (2, 3), treatment modalities (2, 3), timing of assessment (pre- vs. postoperative) (5), and divergent cut-off values (1, 9).

### 4.1 SII cut-off value variation and clinical implications

A critical challenge hindering the clinical application of SII is the substantial heterogeneity in cut-off values across studies, ranging from 340 to 1505 in our analysis (1, 9).Notably, despite the wide variation in cut-off thresholds across studies, our *post-hoc* meta-regression did not identify the SII cut-off value as a significant source of heterogeneity for either OS or PFS, suggesting that threshold variability alone may not explain the observed between-study heterogeneity (**Table 3**). This variability stems from multiple factors: 1) Population differences: Genetic backgrounds, environmental exposures, and baseline inflammatory states vary across geographic regions and ethnic groups; 2) Statistical methodologies: Different approaches for determining optimal thresholds, including receiver operating characteristic (ROC) curve analysis, median values, quartiles, or tertiles; 3) Treatment contexts: SII thresholds may differ between neoadjuvant, adjuvant, and palliative settings; 4) Disease characteristics: Optimal cut-offs may vary between colon and rectal cancers, as evidenced by the different thresholds reported for colon (535) versus rectal (792) liver metastases (7).

This heterogeneity poses significant challenges for clinical implementation and cross-study comparisons. We recommend that future research should: 1) Conduct large-scale prospective multicenter studies using standardized ROC analysis to establish and validate universally applicable optimal cut-off values; 2) Report SII both as continuous variables and categorical variables based on specific thresholds; 3) Consider population-specific reference ranges accounting for age, ethnicity, and geographic factors; 4) Develop dynamic monitoring protocols with standardized time points for SII assessment throughout the treatment trajectory.

### 4.2 Biological mechanisms underlying SII prognostic value

Elevated SII reflects a systemic inflammatory-immune imbalance characterized by: 1) Neutrophilia promoting tumor progression through VEGF and matrix metalloproteinases (MMPs) release, facilitating angiogenesis and metastasis; 2) Thrombocytosis enhancing tumor cell survival in circulation through platelet-tumor aggregate formation, protecting circulating tumor cells from immune surveillance and shear stress; 3) Lymphocytopenia impairing cytotoxic T cell-mediated antitumor immunity, reducing immunosurveillance capacity 4). This inflammatory-immune imbalance contributes to tumor progression by enhancing the infiltration of immunosuppressive cells, including myeloid-derived suppressor cells (MDSCs) and regulatory T cells (Tregs), within the tumor microenvironment.

### 4.3 TNM stage-specific prognostic value

Our comprehensive subgroup analysis by TNM stage revealed important insights: 1) Stage III patients showed the strongest association between elevated SII and poor prognosis (OS: HR = 3.65), suggesting SII may be particularly valuable for risk stratification in this intermediate-stage population where treatment intensification decisions are most critical; 2) Stage I-II patients demonstrated significant associations (OS: HR = 2.31), indicating SII**’**s utility in identifying high-risk early-stage patients who might benefit from adjuvant therapy; 3) Stage IV/metastatic patients showed consistent prognostic value (OS: HR = 2.47), supporting SII**’**s role in treatment monitoring and prognosis prediction in advanced disease.

These findings suggest that SII**’**s prognostic value varies across disease stages, likely reflecting different biological contexts: in early-stage disease, SII may indicate occult micrometastatic burden and systemic inflammatory response to tumor presence; in advanced disease, SII may reflect tumor burden, treatment response, and overall host inflammatory status.

### 4.4 Tumor location heterogeneity

Our analysis by tumor location revealed that SII**’**s prognostic value was consistent across colon, rectal, and mixed colorectal cancers, with HRs of 2.18, 1.96, and 2.05 respectively for OS. However, we acknowledge an important limitation: most original studies did not provide hazard ratios stratified by tumor location, preventing more granular analysis of potential differences between right-sided, left-sided, and rectal cancers. This represents a significant knowledge gap, as tumor location is increasingly recognized as a critical prognostic and predictive factor in CRC.

Future research should prioritize providing location-stratified analyses, as biological differences between proximal and distal CRC may influence SII**’**s prognostic value. Right-sided tumors are associated with different molecular features (higher rates of microsatellite instability, BRAF mutations, and CpG island methylator phenotype), distinct microbiome profiles, and different patterns of systemic inflammation compared to left-sided tumors (4).

### 4.5 Treatment modality and timing considerations

Our analysis revealed important differences based on treatment modality and timing: 1) Post-treatment SII measurements showed stronger associations with prognosis than baseline measurements, suggesting SII**’**s potential as a treatment response biomarker; 2) The attenuated association in patients receiving bevacizumab combination therapy may reflect this agent**’**s anti-inflammatory properties; 3) SII**’**s consistent prognostic value across different treatment modalities supports its broad applicability.

These findings suggest potential clinical applications: 1) Baseline SII assessment for initial risk stratification and treatment planning; 2) Serial SII monitoring during treatment to assess treatment response and detect early progression; 3) Integration with imaging and other biomarkers for comprehensive treatment monitoring. Recent studies suggest that dynamic SII changes (ΔSII) may offer superior prognostic precision compared to static measurements (5).

### 4.6 Geographic bias and generalizability

A significant limitation of our study is the geographic imbalance in included studies, with the majority originating from Asia (particularly China and Japan), limited representation from Europe, and minimal representation from North America, Africa, and other regions. This geographic bias raises important questions about the generalizability of our findings across different populations.

Several factors may contribute to geographic differences in SII prognostic value: 1) Genetic and ethnic variations in inflammatory responses and immune function; 2) Environmental factors including diet, microbiome composition, and exposure to infectious agents; 3) Healthcare system differences affecting cancer screening, diagnosis timing, and treatment protocols; 4) Population-specific baseline inflammatory marker ranges.

We strongly recommend that future research should prioritize inclusion of more diverse, globally representative populations. Multi-center international collaborations are essential to validate SII**’**s prognostic value across different ethnic groups and healthcare systems. Only through such efforts can SII be established as a truly universal prognostic biomarker suitable for global clinical implementation.

### 4.7 Comparison with previous meta-analyses

Our study provides significant incremental value compared to previous meta-analyses, particularly the recent analysis by Tan et al.: 1) Larger sample size: 35 studies with 26812 patients vs. 27 studies with 18,420 patients; 2) More recent literature: search updated to November 2025 vs. March 2024; 3) More comprehensive subgroup analyses: including TNM stage and tumor location analyses not performed in previous studies; 4) More detailed methodological reporting: comprehensive sensitivity analyses and quality assessment.

These enhancements provide stronger evidence for SII**’**s prognostic value and more nuanced understanding of its clinical applications across different patient subgroups.

### 4.8 Limitations and future directions

This study has several limitations requiring cautious interpretation. First, the marked heterogeneity in SII cut-off values across studies may compromise result comparability and clinical applicability. Second, the predominance of retrospective designs (38 of 40 cohorts) introduces potential confounders and selection bias. Third, the geographic imbalance limits generalizability to non-Asian populations. Fourth, insufficient reporting of tumor location-specific data prevented more granular analysis of potential anatomical heterogeneity.

Future research should address these limitations through: 1) Large-scale prospective multicenter studies with standardized protocols; 2) Development and validation of universally applicable SII cut-off values using ROC analysis; 3) Integration of SII with molecular subtyping and other biomarkers for enhanced prognostic accuracy; 4) Investigation of SII**’**s role in treatment selection and monitoring; 5) Exploration of dynamic SII monitoring protocols; 6) Expansion to more geographically diverse populations.

### 4.9 Clinical implementation recommendations

Based on our findings, we propose the following clinical implementation framework: 1) Baseline SII assessment at diagnosis for initial risk stratification; 2) Serial SII monitoring during treatment (e.g., every 3 months) to assess treatment response; 3) Integration with TNM staging to refine prognostic assessment, particularly in Stage III disease; 4) Consideration of treatment intensification for patients with persistently elevated SII; 5) Development of nomograms incorporating SII with other clinical and molecular variables for personalized prognostication.

## 5 Conclusion

This meta-analysis of 35 studies involving 26812 patients demonstrates that elevated pretreatment SII is significantly associated with inferior overall and progression-free survival in colorectal cancer, supporting its utility as a robust prognostic biomarker. The prognostic value is consistent across diverse clinical subgroups, with particularly strong associations in Stage III disease and elderly patients. However, substantial heterogeneity in cut-off values and geographic bias limit immediate clinical implementation.

Large-scale, prospective, multicenter investigations with standardized methodologies are warranted to validate these findings, establish optimal threshold values, and develop evidence-based clinical implementation guidelines. Integrating SII with other biomarkers and molecular subtyping may enhance prognostic accuracy and inform individualized therapeutic strategies in colorectal cancer.

## Data Availability

The original contributions presented in the study are included in the article/**Supplementary Material**. Further inquiries can be directed to the corresponding author.

## Supplementary material

Supplementary Table 1 Literature searching strategy.

Supplementary Table 2 Quality evaluation of the eligible studies with Newcastle–Ottawa scale.
