# Supplementary material for: Prognostic value of systemic immune-inflammation index for colorectal cancer: a systematic review and meta-analysis
Source: Front Oncol. 2026 Feb 5;16:1616016. doi: 10.3389/fonc.2026.1616016 (PMC12916400; doi:10.3389/fonc.2026.1616016)
Supplement: Supplementary Table 1 — Literature searching strategy. [file Table1.docx]

**Supplementary materials**

Table S1 Literature Searching Strategy

**pubmed-142**

(("Colorectal Neoplasms"[Mesh]) OR (((((((((((((((Colorectal Neoplasm) OR (Neoplasm, Colorectal)) OR (Colorectal Tumors)) OR (Colorectal Tumor)) OR (Tumor, Colorectal)) OR (Tumors, Colorectal)) OR (Neoplasms, Colorectal)) OR (Colorectal Cancer)) OR (Cancer, Colorectal)) OR (Cancers, Colorectal)) OR (Colorectal Cancers)) OR (Colorectal Carcinoma)) OR (Carcinoma, Colorectal)) OR (Carcinomas, Colorectal)) OR (Colorectal Carcinomas))) AND ((Systemic Immune-Inflammation Index) OR (SII))

**Embase-105**


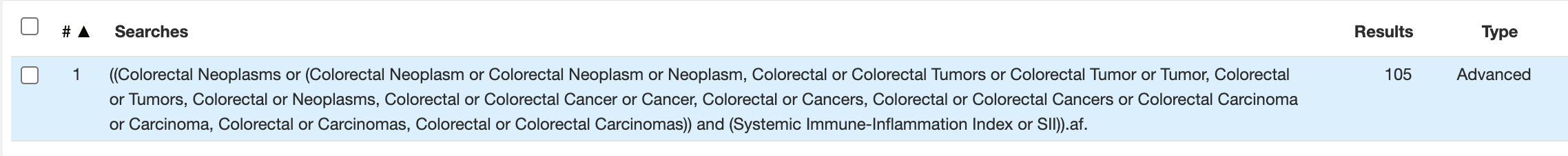


**Cochrane-8**


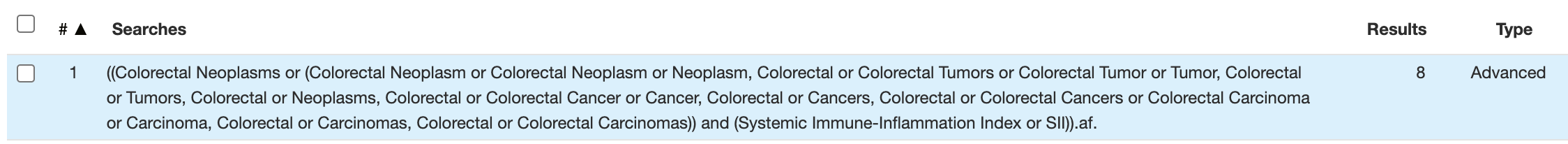


**WOS-94**

((Colorectal Neoplasms) OR (((((((((((((((((Colorectal Neoplasm) ) OR (Colorectal Neoplasm)) OR (Neoplasm, Colorectal)) OR (Colorectal Tumors)) OR (Colorectal Tumor)) OR (Tumor, Colorectal)) OR (Tumors, Colorectal)) OR (Neoplasms, Colorectal)) OR (Colorectal Cancer)) OR (Cancer, Colorectal)) OR (Cancers, Colorectal)) OR (Colorectal Cancers)) OR (Colorectal Carcinoma)) OR (Carcinoma, Colorectal)) OR (Carcinomas, Colorectal)) OR (Colorectal Carcinomas))) AND ((Systemic Immune-Inflammation Index) OR (SII)) (Topic)

Table S2 Quality evaluation of the eligible studies with Newcastle–Ottawa scale

| Study | Selection | | | | Comparability | | Outcome | | |
| --- | --- | --- | --- | --- | --- | --- | --- | --- | --- |
|  | Representative-ness | Selection of non-exposed | Ascertainment of exposure | Outcome not present at start | Comparability on most important factors | Comparability on other risk factors | Assessment of outcome | Long enough follow-up (median≥1 year) | Adequacy (completeness) of follow-up |
| Casadei Gardini 2020 | * | * | * | * | * | - | * | * | * |
| Chang 2023 | * | * | * | * | * | - | * | * | * |
| Chen 2017 | * | * | * | * | * | - | * | * | - |
| Chen 2020 | * | * | * | * | * | - | - | * | * |
| Deng 2021 | * | * | * | * | * | - | * | * | * |
| Gao 2022 | * | * | * | * | * | - | * | - | * |
| Huang 2020 | * | * | * | * | * | * | * | * | * |
| Jiang 2019 | * | * | * | * | * | - | * | * | * |
| Jin 2022 | * | * | * | * | * | - | * | * | * |
| Li 2020 | * | * | * | * | * | - | * | * | * |
| Li 2025 | * | * | * | * | * | - | * | * | * |
| Miyamoto 2023 | * | * | * | * | * | - | * | * | * |
| Moro-Valdezate 2025 | * | * | * | * | * | - | * | * | * |
| Nakamoto 2023 | * | * | * | * | * | - | * | * | * |
| Passardi 2016 | * | * | * | * | * | - | * | * | * |
| Passardi 2023 | * | * | * | * | * | * | * | * | * |
| Polk 2022 | * | * | * | * | * | - | * | * | * |
| Sato 2023 | * | * | * | * | * | - | * | * | * |
| Şentürk 2025 | * | * | * | * | * | - | * | * | * |
| Su 2025 | * | * | * | * | * | - | * | * | * |
| Sun 2024 | * | * | * | * | * | - | * | * | * |
| Xiang 2023 | * | * | * | * | * | * | * | * | * |
| Xie 2018 | * | * | * | * | * | - | * | * | * |
| Xie 2020 | * | * | * | * | * | - | * | * | * |
| Yan 2020 | * | * | * | * | * | - | * | * | * |
| Yang 2017 | * | * | * | * | * | - | * | * | * |
| Yang 2018 | * | * | * | * | * | - | * | * | * |
| Yang 2019 | * | * | * | * | * | - | * | * | * |
| Yatabe 2020 | * | * | * | * | * | - | * | * | * |
| Yi 2023 | * | * | * | * | * | - | * | * | * |
| Young 2023 | * | * | * | * | * | - | * | * | * |
| Yuan 2025 | * | * | * | * | * | - | * | * | * |
| Zeynelgil 2025 | * | * | * | * | * | - | * | * | * |
| Zhang and Miao 2023 | * | * | * | * | * | - | * | * | * |
| Zhou 2018 | * | * | * | * | * | - | * | * | * |

*indicates criterion met; indicates significant of criterion not met.
